# Limited sampling strategy for prolonged-release tacrolimus in renal transplant patients by use of the dried blood spot technique

**DOI:** 10.1007/s00228-015-1863-6

**Published:** 2015-05-17

**Authors:** G. A. J. van Boekel, A. R. T. Donders, K. E. J. Hoogtanders, T. R. A. Havenith, L. B. Hilbrands, R. E. Aarnoutse

**Affiliations:** Department of Nephrology, Radboud university medical center, PO box 9101, 6500 HB Nijmegen, The Netherlands; Department for Health Evidence, Radboud university medical center, PO box 9101, 6500 HB Nijmegen, The Netherlands; Department of Clinical Pharmacy and Toxicology, Maastricht University Medical Centre, PO box 5800, 6202 AZ Maastricht, The Netherlands; School CAPHRI, Maastricht University, PO box 616, 6200 MD Maastricht, The Netherlands; Department of Pharmacy, Radboud university medical center, PO box 9101, 6500 HB Nijmegen, The Netherlands

**Keywords:** Exposure, Limited sampling strategy, Prolonged-release tacrolimus, Renal transplantation

## Abstract

**Purpose:**

The aim of this study was to develop a clinically applicable limited sampling strategy for ambulatory Caucasian kidney transplant patients to estimate area under the curve in a 24-h period (AUC_0–24_) of prolonged-release tacrolimus.

**Methods:**

Twenty six kidney recipients, at least 6 months after transplantation, receiving prolonged-release tacrolimus, were enrolled. In each patient, seven blood samples were collected during a period of 24 h by use of the validated dried blood spot method. Best subset selection multiple linear regression was performed to derive limited sampling strategy (LSS). The equations were constrained to include a maximum of three samples collected within 4 h after the intake to maintain clinical applicability. To assess the predictive performance of LSS, residuals for each patient were calculated based on models fitted to a dataset where that patient was omitted.

**Results:**

The prediction formula for the AUC_0–24_ using the time points 0, 2, and 4 h after ingestion (C_0h_-C_2h_-C_4h_) provided the highest correlation with the AUC_0–24_ (*r*^2^ = 0.95): AUC_0–24_ = 44.9 + 8.9 × C_0h_ + 2.1 × C_2h_ + 7.6 × C_4h_. Measures for bias and precision, i.e., median percentage prediction error (MPPE) and median absolute prediction error (MAPE), were 0.4 and 4.8 %, respectively. For the same patients, the correlation between C_24h_ and AUC_0–24_ was worse (*r*^2^ = 0.77) while MPPE and MAPE were 6.2 and 7.2 %, respectively.

**Conclusion:**

In the outpatient department, a LSS using C_0h_-C_2h_-C_4h_ can be used for reliable estimation of the AUC_0–24_ of prolonged-release tacrolimus_._

**Electronic supplementary material:**

The online version of this article (doi:10.1007/s00228-015-1863-6) contains supplementary material, which is available to authorized users.

## Introduction

Tacrolimus is a usual component of the immunosuppressive regimen after renal transplantation. While it was developed as an oral twice-daily formulation, a prolonged-release once-daily formulation was launched a few years ago. The efficacy and safety profile of prolonged-release tacrolimus are comparable to that of the twice-daily formulation [3, 9]. Kuijpers et al. demonstrated that the once-daily administration of tacrolimus improves adherence, which might ultimately contribute to better graft outcomes [11]. Moreover, intra patient variability in exposure is somewhat lower with the prolonged-release formulation [18].

Although the relationship between tacrolimus exposure and clinical response has not yet been fully established, therapeutic drug monitoring (TDM), i.e., individualization of the dose based on concentration measurements, is indicated for tacrolimus. TDM aims to improve the efficacy of tacrolimus, prevents overexposure and associated adverse effects, and detects drug interactions or unexpected pharmacogenetic influences on exposure to this immunosuppressive drug [21]. In an expert meeting, it was concluded that the area under the concentration versus time curve (AUC), which is calculated on the basis of a full pharmacokinetic profile, is the best measure of exposure to tacrolimus [21]. However, assessment of a full pharmacokinetic profile requires the collection of many blood samples and is therefore costly, time consuming, and uncomfortable, in particular for ambulatory patients. These drawbacks hinder recording of the AUC in routine practice. Trough levels are commonly used to estimate exposure since they show a moderate to high correlation with AUC [2, 3, 6, 20, 23]. In spite of this moderate to high correlation, AUC can vary up to twofold for the same trough level. For that reason, concerns have been raised about the use of the trough level [5].

A more reliable estimation of the total exposure can be obtained by a limited sampling strategy (LSS). This means sampling at limited or optimal sampling times, still allowing for an accurate and precise estimation of the AUC [15, 19]. Clearly, a LSS also overcomes logistical and financial disadvantages of a full pharmacokinetic profile. However, an appropriate LSS for ambulatory Caucasian renal transplant patients who use prolonged-release tacrolimus is not available. Therefore, the aim of this study was to develop a clinically applicable LSS to estimate the area under the curve in a 24-h period (AUC_0–24_) of prolonged-release tacrolimus in them. This would also allow us to assess the performance of the trough level, as a single sample, to predict the AUC_0–24_ of prolonged-release tacrolimus.

## Patients and methods

### Study design and population

We carried out a prospective pharmacokinetic study to assess tacrolimus concentrations during a period of 24 h after ingestion of prolonged-release tacrolimus in the morning.

Adult renal transplant patients with a stable graft function were eligible for enrolment if they used prolonged-release tacrolimus (Advagraf®, Astellas Pharma) of which the dose was not altered during the last visit to the outpatient clinic, and the two most recently measured trough levels were within the target range of 5–10 μg/L. Patients were excluded if they were unable to perform the home-based dried blood spot measurements of tacrolimus levels (see below) or if they had diarrhea (more than three stools per day) during the preceding 14 days, as we considered that diarrhea might affect the ratio between AUC_0–24_ and trough levels [12].

The study was approved by the local ethics committee and conducted in accordance with the 1964 Helsinki Declaration and its later amendments. Informed consent was obtained from all individual participants included in the study.

### Measurements

A validated dried blood spot method for sampling and analysis of tacrolimus was used, which allowed participants to take their own blood samples at home [10]. Accuracy and intra- and interassay precision were <15 and <7.5 %, respectively. Using this method, capillary blood is obtained by a finger prick with an automatic lancet by the patients themselves. Subsequently, the first two drops of blood are applied to the sampling paper to fill two 8-mm premarked circles for duplicate sampling. After at least 10 min drying at room temperature, the samples are stored in a sealed plastic bag and sent by regular post to the laboratory. Here, the disks from the blood spot are punched out, extracted, and analyzed by a specific high-performance liquid chromatography-tandem mass spectrometry (HPLC-MS/MS) method. Participants received thorough training in using this method prior to performing the pharmacokinetic measurements, and they could only be included if their test blood sample passed the quality control.

Each pharmacokinetic profile started with measurement of the whole blood tacrolimus concentration at 24 h after the previous morning ingestion of prolonged-release tacrolimus and after overnight fasting (C_0_). Subsequently, prolonged-release tacrolimus was taken and blood samples were collected at 1, 2, 4, 8, 12, and 24 h after the ingestion. On the day of the measurements, all participants took prolonged-release tacrolimus on an empty stomach and refrained from food intake until 2 h after its ingestion since it has been reported that the tacrolimus concentration profile can be influenced by meal consumption [7]. Administration of prolonged-release tacrolimus on an empty stomach is also recommended in the product information.

Pharmacokinetic parameters were assessed with noncompartmental methods using WinNonLin version 5.0 (Pharsight Co version 5.0, Mountain View, CA). The AUC_0–24_ was calculated by the linear-log trapezoidal rule. C_0_, C_24_, the maximum blood concentration (*C*_max_), and time required to reach it (*T*_max_) were directly read from the pharmacokinetic curves. Elimination rate constant *β* was obtained by least squares linear regression analysis on logarithmic concentrations versus time post-dose, with the slope of the regression line being *-β*/2.303. Apparent clearance (Cl/*F* where *F* is bioavailability) was calculated by dividing dose by AUC_0–24_ and apparent volume of distribution (Vd/*F*) was obtained by dividing Cl/*F* by *β*.

### Statistical analysis

Loose concentrations and pharmacokinetic parameters were described with a geometric mean and 95 % confidence interval. *T*_max_ and daily tacrolimus dose were presented as median and range. The other data were shown as median with interquartile range. Correlations between numerical variables were calculated using the non-parametric Spearman’s rank correlation coefficient (rho).

We carried out best subset selection multiple linear regression to derive limited sampling equations which predicted the AUC_0–24_. Models were constrained to include a maximum of three samples collected within 4 h after the intake to maintain clinical applicability for patients in the outpatient department. We also developed models with a maximum of three samples within 12 h after the intake since some patients execute home-based measurements by use of the dried blood spot technique. As they do not need to stay in the outpatient department for the measurements, the time span of the LSS is less relevant. We calculated the average adjusted r square for all subsets containing one, two, or three samples, and the model with the highest average r square was chosen.

To assess the predictive performances of the models, we calculated residuals for each patient based on models fitted to a dataset where that patient was omitted (jackknife analysis) [19]. Potential bias in the predictions was assessed by using median percentage prediction error (MPPE). For this measure, residuals were converted to percentages by dividing the residuals by the predicted values. We considered MPPE < 5 % to be acceptable. Imprecision was assessed using median absolute percentage prediction error (MAPE) for which we accepted a percentage limit of <10 %. These strict criteria for predictive performance were chosen considering the narrow therapeutic index of tacrolimus, for which small changes in total exposure could be clinically relevant. This is also reflected in altered European guidelines for drug formulation bio-equivalence studies, specifying that for narrow therapeutic index drugs the acceptance interval for AUC can be tightened to 90.0–111.1 % [1]. Niioka et al. recently used the same criteria for MPPE and MAPE to evaluate their LSS for prolonged-release tacrolimus in Japanese renal transplant recipients [13].

All statistical analyses were performed with SPSS for Windows version 20.0 (SPSS Inc, Chicago, IL) and R version 2.15.2. *P* values of below 0.05 were considered significant.

## Results

Of the thirty patients who were recruited for the study, 26 patients completed a full pharmacokinetic profile. Four exclusions were due to insufficient quality of the blood spots (*n* = 1), and giving up informed consent (*n* = 3). All 26 participants had an isolated kidney transplantation and were of Caucasian ancestry. Other patient characteristics are shown in Table 1.

**Table 1:** Patient characteristics (*n* = 26)

Male (%)	69
Caucasian (%)	100
Age (years)	43.9 (36.1–57.6)
Weight (kg)	78.4 (72.6–86.1)
Time after transplantation (years)	5.4 (2.3–7.1)
eGFR (mL/min/1.73 m^2^)	51.5 (40.8–59.3)
Hematocrit	0.39 (0.36–0.41)
Albumin (g/L)	39.5 (36.0–41.0)
Use of calcium channel blockers (%)	58
Use of steroids (%)	77

Data are shown as median with interquartile range

The median daily dose of prolonged-release tacrolimus was 4.0 mg (range 1.5–10.0). The geometric mean C_0_ and 24-h post-dose trough level (C_24_) were 7.7 μg/L (95 % confidence interval 7.0–8.5) and 8.3 μg/L (7.5–9.1), respectively. The geometric mean AUC_0–24_ of tacrolimus was 288 μg h/L (262–317). The mean ratio of AUC_0–24_ and C_24_ was 34.8 (33.2–36.5). The pharmacokinetics of prolonged-release tacrolimus are summarized in Supplementary Figure 1 and Supplementary Table 1.

Various sampling time points and combinations of sampling time points were used to construct a regression model for the prediction of the AUC_0–24_. Relatively high adjusted *r*^2^ values, approaching 0.90, could already be reached with inclusion of only one sampling time, but *r*^2^ increased with the inclusion of two or three sampling times. The best performing limited sampling strategies for ambulatory patients in the outpatient department, based on one, two, or three time points within 4 h post-dose, are shown in Table 2A. Sampling at 0, 2, and 4 h post-dose provided the best prediction of AUC_0–24._ The adjusted *r*^2^ was 0.95 and MPPE and MAPE, measures for bias and precision of the prediction formulas, were 0.4 and 4.8 %, respectively. For patients who performed home-based measurements (sampling allowed up to 12 h post-dose), an equation with sampling times at 2, 8, and 12 h post-dose revealed the best results. The adjusted *r*^2^ was 0.98 and MPPE and MAPE were 0.4 and 2.8 %, respectively (Table 2B).

**Table 2:** Best performing one, two and three sampling strategies for estimation of AUC_0–24_ of prolonged-release tacrolimus for (A) sampling up to 4 h allowed, and (B) sampling up to 12 h allowed

Sampling times	Equation	Adjusted *r* ^2^	MPPE %	MAPE %
A				
T_4_	AUC_0–24_ = 107.6 + 10.6 × C_4_	0.89	−0.25	5.73
T_0_, T_4_	AUC_0–24_ = 57.5 + 10.1 × C_0_ + 8.9 × C_4_	0.93	0.45	5.27
T_0_, T_2_, T_4_	AUC_0–24_ = 44.9 + 8.9 × C_0_ + 2.1 × C_2_ + 7.6 × C_4_	0.95	0.38	4.80
B				
T_8_	AUC_0–24_ = 37.6 + 20.6 × C_8_	0.89	1.23	5.21
T_8_, T_12_	AUC_0–24_ = −8.8 + 11.8 × C_8_ + 14.2 × C_12_	0.96	−0.28	4.71
T_2_, T_8_, T_12_	AUC_0–24_ = −12.8 + 2.1 × C_2_ + 9.7 × C_8_ + 12.7 × C_12_	0.98	−0.38	2.82

The equations were developed for Caucasian kidney transplant recipients who took prolonged-release tacrolimus on an empty stomach

*MPPE* median percentage prediction error, *MAPE* median absolute prediction error, *AUC*_*0–24*_ area under the curve in a 24-h period

The commonly used trough level (C_24_) to predict exposure to prolonged-release tacrolimus provided an adjusted *r*^2^ of 0.77 with AUC_0–24_. MPPE and MAPE of C_24_ were 6.2 and 7.2 %, respectively. The strongest correlation between a single measurement and AUC_0–24_ was found for C_8_ (adjusted *r*^2^ = 0.89) while MPPE and MAPE in this case were 1.2 and 5.2 % (Table 2B).

No relevant effect of hematocrit and co-administration of steroids and calcium channel blockers (CCBs) on adjusted *r*^2^ and predictive performances of the prediction formulas with sampling at 0, 2, and 4 h or at 2, 8, and 12 h post-dose was apparent (Supplementary Table 2).

## Discussion

In this study, we developed equations to estimate AUC_0–24_ of prolonged-release tacrolimus which are clinically applicable for ambulatory Caucasian renal transplant patients. These strategies enclose up to three sampling time points and provide an accurate and precise estimation of the AUC_0–24_.

In daily clinical practice, the trough level (C_24_) of prolonged-release tacrolimus is commonly used to establish whether exposure is adequate. The dose of prolonged-release tacrolimus is adjusted if C_24_ is outside the defined target range. According to most studies, there is a moderate to high correlation between trough level and exposure to prolonged-release tacrolimus [2, 3, 6, 20, 23]. In fact, *r*^2^ of 0.77 in the current study confirms this correlation. However, concerns have been raised that this degree of correlation is not sufficient since correlation is not a good measure for predictive performance [5]. It should be better evaluated in terms of bias and precision, which are expressed as MPPE and MAPE, respectively [16, 19]. Criteria for MPPE and MAPE are somewhat arbitrary and maximum values of 15–20 % have been suggested before [19]. However, these limits are not strict enough for a drug with a narrow therapeutic index. In agreement with Niioka et al., we chose to use 5 and 10 % as cut-off values for acceptable MPPE and MAPE [13]. According to these criteria, the prediction of AUC_0–24_ based on trough levels was biased (yet precise). Interestingly, Scholten et al. showed that overexposure to tacrolimus in patients using the regular formulation was less frequent when dosing was based on AUC measurements as compared to trough levels only [15]. Although this has not been demonstrated for the prolonged-release formulation of tacrolimus, it is likely that the same holds true here since the correlation between trough levels and AUC are about the same for either formulation.

The two main approaches for developing LSS are multiple linear regression and Bayesian analysis. They are equally valid but have different advantages and disadvantages [19]. We preferred multiple regression analysis as it is relatively simple to develop, to use, and to incorporate in clinical practice. The regression analyses are conceptually clear, and this approach is not dependent on knowledge or assumptions on the pharmacokinetics of the drug and the choice of a specific pharmacokinetic model. Once equations are obtained, AUC values can be estimated by straightforward calculations that can even be done manually. Disadvantages are that only limited deviation from target sampling times is allowed and that the LSS is only applicable for patients with similar characteristics as for whom the strategy was developed. Our LSS is therefore only applicable in Caucasian patients who take prolonged-release tacrolimus on an empty stomach as recommended in the product information. The Bayesian forecasting method uses population pharmacokinetic data, drug dosing information, and measured concentrations to estimate pharmacokinetic parameters of the individual patient. Advantages of Bayesian forecasting combined with limited sampling are flexibility with sampling times, and simultaneous prediction of several pharmacokinetic parameters. Moreover, the LSS can continuously be updated by incorporating new data into the population data set. However, the Bayesian strategy also requires expertise with specific software and a choice for a pharmacokinetic model of the drug that affects the predictive performance of the LSS. Furthermore, users need extensive training in operating the programs and interpreting the results, and Bayesian analysis demands for rather extensive data entry which may not be feasible in daily clinical practice.

In our study, we did not prospectively validate our limited sampling formulas. Alternatively, we computed residuals for every patient in a model which fitted to a dataset where that patient was omitted. This analysis is a useful and appropriate approach if the sample size is small [19].

Differences in pharmacokinetics of tacrolimus are partly explained by genetic polymorphisms of CYP3A5 [17]. Moreover, co-medication as steroids and CCBs affect the pharmacokinetics of this drug. Therefore, it is important to determine whether bias and precision in the several prediction formulas are affected by CYP3A5 polymorphism or use of co-medication. We showed that CYP3A5 polymorphisms and co-medication did not significantly influence the performances of the prediction formulas. Niioka et al. previously showed that in their developed LSS, the effect of CYP3A5 polymorphism was also irrelevant [13].

One LSS using multiple linear regression and three limited sampling strategies by use of the Bayesian forecasting method have currently been published for prolonged-release tacrolimus in adult renal transplant patients [5, 13, 14, 24]. The LSS for prolonged-release tacrolimus which used multiple regression was described by Niioka et al. [13]. Their LSS was developed in Japanese patients while our LSS is applicable for Caucasian patients. Moreover, they used the chemiluminescence magnetic microparticle immunoassay on the Architect-i1000 system (Abbott Laboratories; Abbott Park, IL) for bio-analysis of tacrolimus, whereas we used the highly specific HPLC-MS/MS method which does not measure cross-reacting inactive tacrolimus metabolites [4, 8, 22]. For limited sampling during a period of 4 h, they also found 0, 2, and 4 h post-dose as optimal sampling strategy. However, the correlation between predicted and observed AUC_0–24_ appeared higher (*r*^2^ 0.95 versus 0.85) and precision appeared better (MAPE 4.8 versus 9.8 %) with our formula. Their LSS for home-based measurements contained 3 sampling time points at 0, 3, and 12 h post-dose, compared to 2, 8, and 12 h post-dose in our study. The correlation and predictive performances appeared comparable. The limited sampling strategies using the Bayesian forecasting method all enclosed sampling at 0, 1, and 3 h post-dose [5, 14, 24].

For this study, we used the innovative “dried blood spot” technique which has proven to be very reliable for determining tacrolimus levels [10]. This technique requires careful training of the patients involved, but then obviates the need for hospital visits, phlebotomies, and clinical research facilities. Therefore, this technique seems very suitable for observational pharmacokinetic studies in a home-based, day-to-day clinical setting [18]. In routine clinical care, the dried blood spot technique can also be used for a selected group of patients who can be taught well and are able to execute it. Therefore, we also developed prediction formulas in which the time span of LSS is less relevant.

Finally, the AUC of tacrolimus is considered to be a better measure for exposure than the trough level [21]. However, it has not been proven that AUC-based TDM resulted in a better graft or patient survival than trough level-based TDM [21]. Moreover, the target AUC of tacrolimus for patients who are more than 6 months after renal transplantation has to be established. Additional prospective trials with large patient numbers would therefore be needed to assess the effects of using AUC versus trough level on outcome and to determine the target AUC. We would especially be interested in studies with patients for whom a better estimation of the exposure to tacrolimus is critical, like highly immunized patients or patients with calcineurin inhibitor nephrotoxicity.

In conclusion, a LSS using samples collected before the intake and at 2 and 4 h post-dose can be applied for a very accurate and precise estimation of the AUC_0–24_ of prolonged-release tacrolimus in ambulatory renal transplant patients. A LSS with samples obtained before the intake and at 3 and 12 h also showed a good predictive performance but it seems only suitable for patients who can execute the dried blood spot measurement at home.

## Electronic supplementary material

Supplementary Figure 1 Tacrolimus concentration-time curve. Data are shown as geometric mean with 95 % confidence interval. (JPEG 215 kb)

Supplementary Table 1 (DOCX 11 kb)

Supplementary Table 2 (DOCX 11 kb)
