# Supplementary figures and images for: Limited sampling strategy for prolonged-release tacrolimus in renal transplant patients by use of the dried blood spot technique
Source: Eur J Clin Pharmacol. 2015 May 17;71(7):811–6. doi: 10.1007/s00228-015-1863-6 (PMC4464598; doi:10.1007/s00228-015-1863-6)

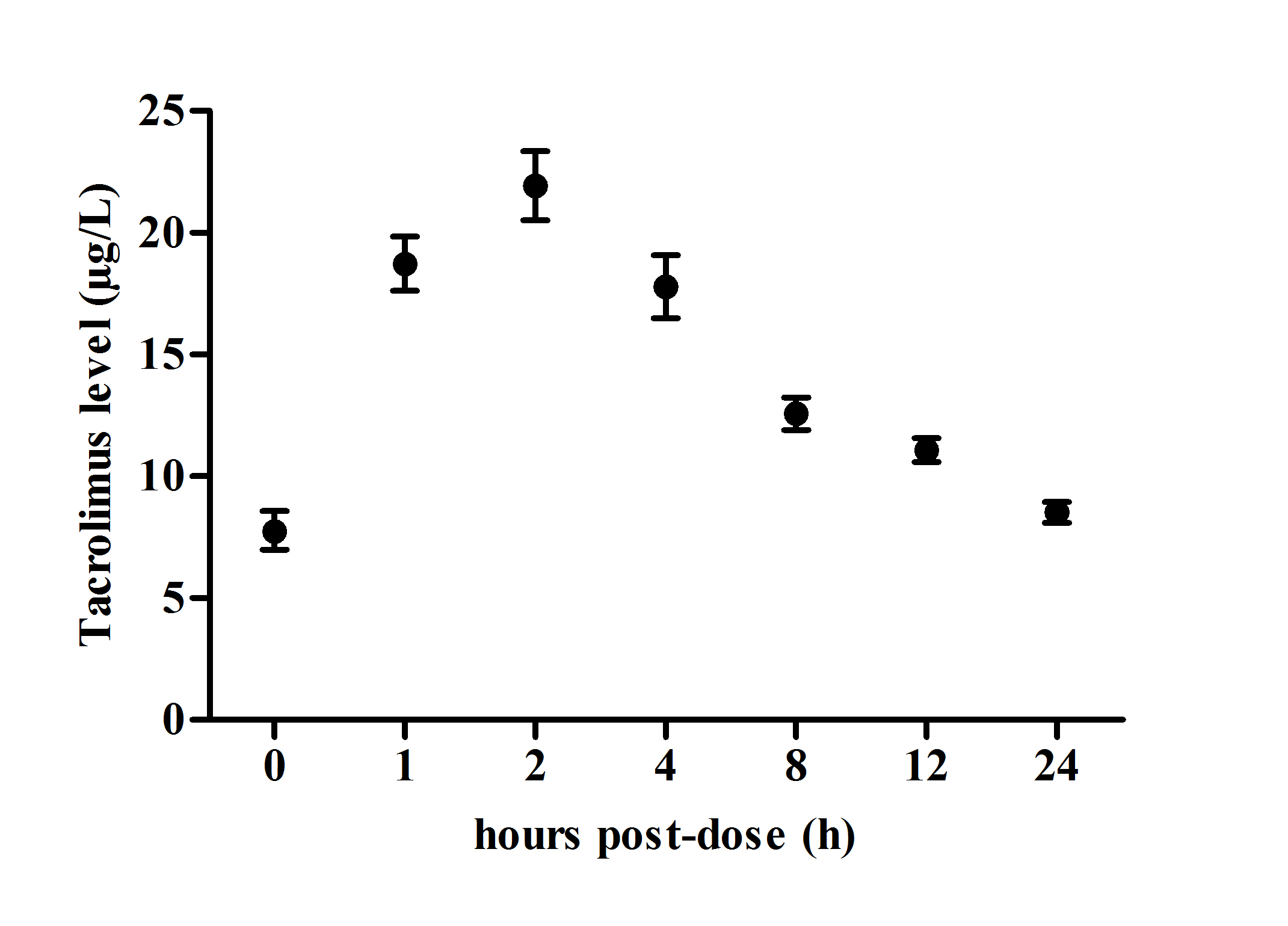

Supplement: Supplementary file 1 — Tacrolimus concentration-time curve. Data are shown as geometric mean with 95 % confidence interval. (JPEG 215 kb) [file 228_2015_1863_Fig1_ESM.jpg]
