# Supplementary material for: Limited sampling strategy for prolonged-release tacrolimus in renal transplant patients by use of the dried blood spot technique
Source: Eur J Clin Pharmacol. 2015 May 17;71(7):811–6. doi: 10.1007/s00228-015-1863-6 (PMC4464598; doi:10.1007/s00228-015-1863-6)
Supplement: Supplementary file 2 — (DOCX 11 kb) [file 228_2015_1863_MOESM1_ESM.docx]

**Supplementary Table 1. Pharmacokinetic parameters (n=26)**

| **AUC_(0-24)_ (µg.h/L)** | 288 (262-317) |
| --- | --- |
| **C_max_ (µg/L)** | 22.1 (19.4-25.2) |
| **T_max_ (h)** | 2.0 (1.0-4.1) |
| **T_½_ (h)** | 29.4 (26.3-33.0) |
| **Cl/F (L/h)** | 15.2 (12.9-18.0) |
| **Vd/F (L)** | 647(545-768) |

^Data are shown as geometric mean (95% confidence interval) except in the case of Tmax, which is shown as median and range. Cmax: maximal concentration; AUC(0-24): area under the curve in a 24 hours period; Tmax: time for reaching Cmax; T½: elimination half time; Cl/F: apparent clearance; Vd/F: apparent volume of distribution. Intake of prolonged-release tacrolimus was on an empty stomach.^
