# Supplementary material for: Limited sampling strategy for prolonged-release tacrolimus in renal transplant patients by use of the dried blood spot technique
Source: Eur J Clin Pharmacol. 2015 May 17;71(7):811–6. doi: 10.1007/s00228-015-1863-6 (PMC4464598; doi:10.1007/s00228-015-1863-6)
Supplement: Supplementary file 3 — (DOCX 11 kb) [file 228_2015_1863_MOESM2_ESM.docx]

**Supplementary Table 2. Predictive performance of the equations in subgroups**

|  | | **Sampling T_0_,T_2_,T_4_** | | **Sampling at T_2_,T_8_,T_12_** | |
| --- | --- | --- | --- | --- | --- |
|  |  | MPPE | MAPE | MPPE | MAPE |
| **Steroids** | using (n=20) | -1.2% | 4.3% | 1.3% | 2.4% |
|  | not using (n=6) | 2.5% | 4.7% | -3.0% | 2.9% |
| **CCBs** | using (n=15) | -0.4% | 5.0% | 1.3% | 2.7% |
|  | non using (n=11) | -0.3% | 3.6% | -1.1% | 2.4% |

^Abbreviations: MPPE: median percentage prediction error; MAPE: median absolute prediction error; CCBs: calcium channel blockers.^
